# Quality of Computationally Inferred Gene Ontology Annotations

**DOI:** 10.1371/journal.pcbi.1002533

**Published:** 2012-05-31

**Authors:** Nives Škunca, Adrian Altenhoff, Christophe Dessimoz

**Affiliations:** 1 Ruđer Bošković Institute, Division of Electronics, Zagreb, Croatia; 2 ETH Zurich, Computer Science, Zurich, Switzerland; 3 Swiss Institute of Bioinformatics, Zurich, Switzerland; 4 EMBL-European Bioinformatics Institute, Hinxton, Cambridge, United Kingdom; NNF Center for Protein Research, Denmark

## Abstract

Gene Ontology (GO) has established itself as the undisputed standard for protein function annotation. Most annotations are inferred electronically, i.e. without individual curator supervision, but they are widely considered unreliable. At the same time, we crucially depend on those automated annotations, as most newly sequenced genomes are non-model organisms. Here, we introduce a methodology to systematically and quantitatively evaluate electronic annotations. By exploiting changes in successive releases of the UniProt Gene Ontology Annotation database, we assessed the quality of electronic annotations in terms of specificity, reliability, and coverage. Overall, we not only found that electronic annotations have significantly improved in recent years, but also that their reliability now rivals that of annotations inferred by curators when they use evidence other than experiments from primary literature. This work provides the means to identify the subset of electronic annotations that can be relied upon—an important outcome given that >98% of all annotations are inferred without direct curation.

## Author Summary

In the UniProt Gene Ontology Annotation database, the largest repository of functional annotations, over 98% of all function annotations are inferred *in silico*, without curator oversight. Yet these “electronic GO annotations” are generally perceived as unreliable; they are disregarded in many studies. In this article, we introduce novel methodology to systematically evaluate the quality of electronic annotations. We then provide the first comprehensive assessment of the reliability of electronic GO annotations. Overall, we found that electronic annotations are more reliable than generally believed, to an extent that they are competitive with annotations inferred by curators when they use evidence other than experiments from primary literature. But we also report significant variations among inference methods, types of annotations, and organisms. This work provides guidance for Gene Ontology users and lays the foundations for improving computational approaches to GO function inference.

## Introduction

Gene Ontology (GO) annotations are a powerful way of capturing the functional information assigned to gene products [1]. The organization of the GO in a Directed Acyclic Graph allows for various levels of assignment specificity, while the three ontologies—Biological Process, Molecular Function, and Cellular Component—capture three aspects of the gene product annotation.

**Figure 1:**
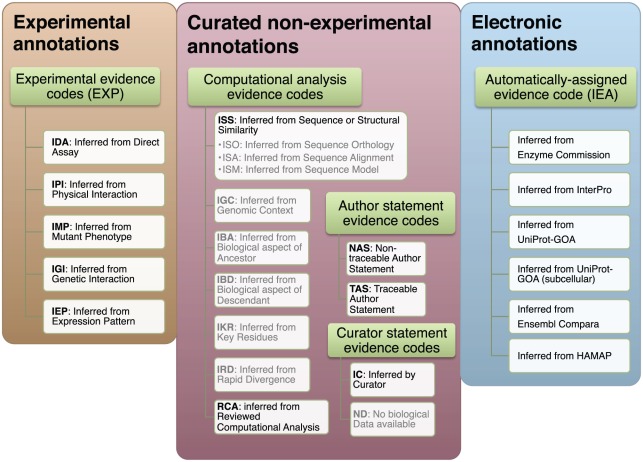
A list of the Gene Ontology (GO) evidence and reference codes we analyzed. We group the GO evidence codes in three groups: experimental, non-experimental curated, and electronic. Gray text denotes the evidence codes that were not included in the analysis: they are either used to indicate curation status/progress (ND), are obsolete (NR), or there is not enough data to make a reliable estimate of their quality (ISO, ISA, ISM, IGC, IBA, IBD, IKR, IRD). The subdivision of the evidence codes (green rectangles) reflects the subdivision available in the GO documentation: http://www.geneontology.org/GO.evidence.shtml.

Some GO annotations are assigned by expert curators, either from experimental evidence in the primary literature (*experimental* annotations), or from other evidence such as sequence similarity, review papers and database entries (*curated* annotations). However, the vast majority (>98%) of available GO annotations are assigned using computational methods, without curator oversight [2] (Fig. 1).

Uncurated—*electronic*—annotations are generally considered to be least reliable. Many users of GO annotations err on the safe side by assigning a lower rank/weight to electronic annotations or leave them completely out of their analyses [e.g.], [ 3]–[7]. However, there have been very few evaluations of the quality of electronic annotations. To our knowledge, the most relevant study to date assessed the annotation quality of only 286 human proteins [8].

Here, we provide the first comprehensive evaluation of electronic GO annotation quality. Based on successive releases of the UniProt Gene Ontology Annotation database (UniProt-GOA), the largest contributor of electronic annotations [9], we used experimental annotations added in newer releases to confirm or reject electronic annotations from older releases. We defined 3 measures of annotation quality for a GO term: 1) *reliability* measures the proportion of electronic annotations later confirmed by new experimental annotations, 2) *coverage* measures the power of electronic annotations to predict experimental annotations, and 3) *specificity* measures how informative the predicted GO terms are.

After describing our new methodology in detail, we first consider changes in quality in UniProt-GOA over time. We then characterize the relationship between GO term reliability and specificity. Next, we consider possible differences in quality among the three ontologies, among computational methods used to infer the electronic annotations, and among the 12 best-annotated model organisms. Finally, we contrast electronic annotations with curated annotations that use evidence other than experiments from primary literature.

## Results

**Figure 2:**
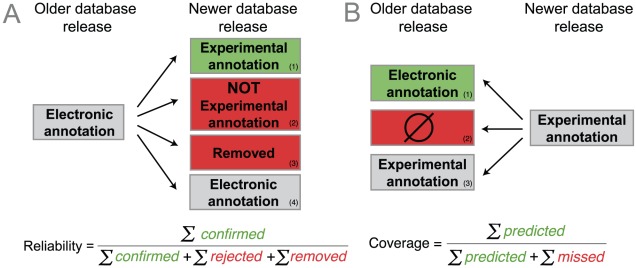
Outline of the strategy to evaluate electronic Gene Ontology annotations. (**A**) *Reliability* measures the proportion of electronic annotations confirmed by future experimental annotations: an electronic annotation in an older database release is either 1) confirmed by a new experimental annotation in the later release, 2) falsified by a new, contradictory experimental annotation (corresponding GO term, but with ‘NOT’ qualifier, which amounts to an explicit rejection), 3) removed from the new UniProt-GOA release (implicit rejection), or 4) unchanged, which is uninformative and does not affect the reliability measure. (**B**) *Coverage* measures the extent to which electronic annotations can predict future experimental annotations: an experimental annotation in the newer release is either 1) correctly predicted by an electronic annotation in the older release, or 2) not correctly predicted (“missed”). Note that the strategy is outlined for electronic annotations, but any subset of annotations can be analyzed this way, e.g. annotations assigned using a selection of evidence or reference codes.

To evaluate the quality of electronic annotations, we tracked changes in UniProt Gene Ontology Annotation (UniProt-GOA) database releases in overlapping three-year intervals. As a surrogate for the intuitive notion of correctness, we define the *reliability* as the ratio of confirmed electronic annotations to confirmed and rejected/removed ones. One electronic annotation is deemed confirmed or rejected, depending on whether a new, corresponding experimental annotation supports or contradicts it. Furthermore, if an electronic annotation is removed, the annotation is deemed implicitly rejected and thus contributes negatively to the reliability measure (Fig. 2 A). As a surrogate for the intuitive notion of sensitivity, we define *coverage* as the proportion of newly added experimental annotations that had been correctly predicted by an electronic annotation in a previous release (Fig. 2 B).

The addition of new experimental annotations—high-quality annotations assigned by a curator—allows us to evaluate the existing electronic annotations. Unfortunately, the set of available experimental annotations is small, since obtaining them requires valuable curator time. Moreover, resource constraints require that curators focus their efforts on a selected set of model organisms [10]. Consequently, most of the available experimental annotations are distributed among the model organisms (Fig. S1 in Text S1); it is this set of genomes that we analyze.

### Electronic annotations in subsequent UniProt-GOA releases are increasing in quality

**Figure 3:**
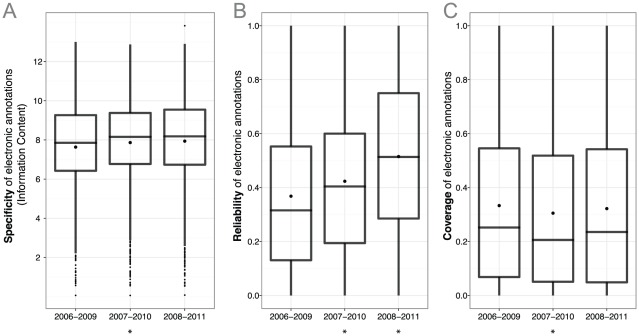
Summary statistics of GO terms: (A) specificity, (B) reliability, and (C) coverage. Each boxplot summarizes the measure of quality indicated on the y-axis for the evaluation period indicated on the x-axis. Lower, mid, and upper horizontal lines denote the first quartile, median and the third quartile, respectively, while the black dots denote the mean values. Outliers (further than 1.5 interquartile range from the respective quartile) are denoted by black points. An asterisk (*) below the boxplot denotes a significant difference of the median with respect to the previous interval, at a confidence level of 0.05 (Mann-Whitney U test, two-tailed).

We first sought to evaluate general trends in the overall quality of UniProt-GOA. Four summary statistics—first and third quartile, median, and mean—allow us to describe the change in quality—specificity, reliability, and coverage—of successive UniProt-GOA releases (Fig. 3). Subsequent UniProt-GOA releases are improving with the addition of slightly more specific annotations on average (Fig. 3 A). At the same time, new UniProt-GOA releases show steady and significant improvement in reliability, as indicated by the increase of all four summary statistics (Fig. 3 B). By contrast, the coverage of annotations has decreased somewhat (Fig. 3 C). Taken together, these indicators suggest a general improvement in the quality of recent UniProt-GOA releases.

### GO term's specificity is only partially indicative of the reliability of electronic annotations

**Figure 4:**
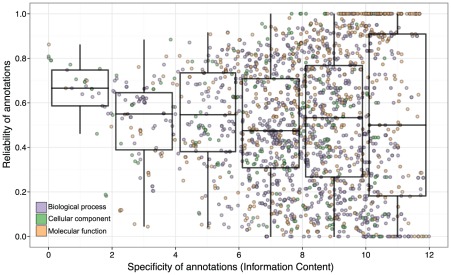
Reliability of electronic annotations in the 16-01-2008 UniProt-GOA release compared to the specificity of the assigned GO term—Information Content in the 16-01-2008 UniProt-GOA release. Each point represents one GO term, and its color corresponds to the ontology in the legend. Each boxplot summarizes the reliability of a selection of GO terms: those with specificity in the range denoted by the width of the boxplot. Lower, mid, and upper horizontal lines denote the first quartile, median and the third quartile, respectively. Vertical lines reach the 1.5 interquartile ranges from the respective quartiles or reach the extreme value, whichever is closer. To be visualized in these plots, a GO term needs to have assigned at least 10 electronic annotations in the 16-01-2008 UniProt-GOA release and at least 10 experimental annotations in the 11-01-2011 UniProt-GOA release.

Next, we investigated the association between a GO term's specificity and reliability (Fig. 4). Previous works based on smaller datasets have observed a negative relation between the predictive power of computational annotation and the specificity of the assigned GO term [e.g.], [ 11]–[13]. Our results are consistent with these results to the extent that almost all general terms are stable (Fig. 4). Specific terms, however, span the whole range of reliability. We also observe that on average, reliability of electronic annotations hardly depends on their specificity: the variance of reliability increases with an increase in specificity, but the median stays largely constant.

### The three ontologies have similar reliability, but different coverage

**Figure 5:**
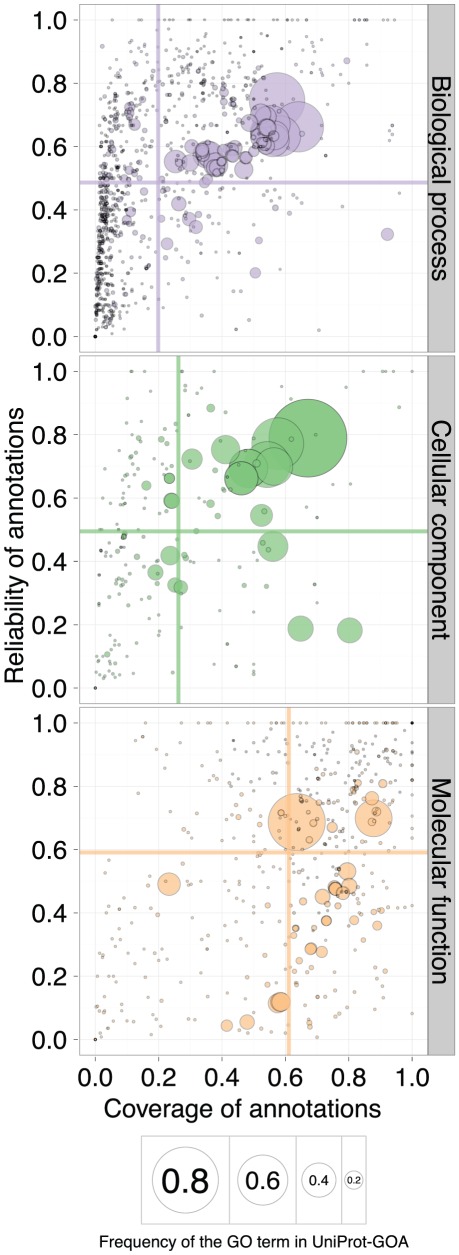
The quality of the 16-01-2008 UniProt-GOA release, evaluated by the 11-01-2011 UniProt-GOA release. A scatterplot of coverage compared to the reliability for the GO terms of the three ontologies: Biological Process, Cellular Component, and Molecular Function. The area of the disc reflects the frequency of the GO term in the 16-01-2008 UniProt-GOA release. The colored lines correspond to the mean values for the respective axes. To be visualized in this plot, a GO term needs to have assigned at least 10 electronic annotations in the 16-01-2008 UniProt-GOA release and at least 10 experimental annotations in the 11-01-2011 UniProt-GOA release. An interactive plot is available at http://people.inf.ethz.ch/skuncan/SupplementaryVisualization1.html.

To assess the differences in annotation quality among the three ontologies, we analyzed the ontologies separately in terms of reliability, coverage, and specificity. On average, annotations associated with the three ontologies were similarly stable, but vary considerably in coverage (Fig. 5). Specifically, Biological Process (BP) terms had the lowest coverage, Molecular Function (MF) terms had the highest coverage, and Cellular Component (CC) terms were in-between. This is consistent with the notion that MF terms are easiest to assign, and BP terms hardest to assign [14]. Nevertheless, this difference in difficulty translates into variable coverage but very similar reliability, suggesting that the false-positive rate of electronic annotations is controlled effectively.

### Different sources provide annotations of different quality

**Figure 6:**
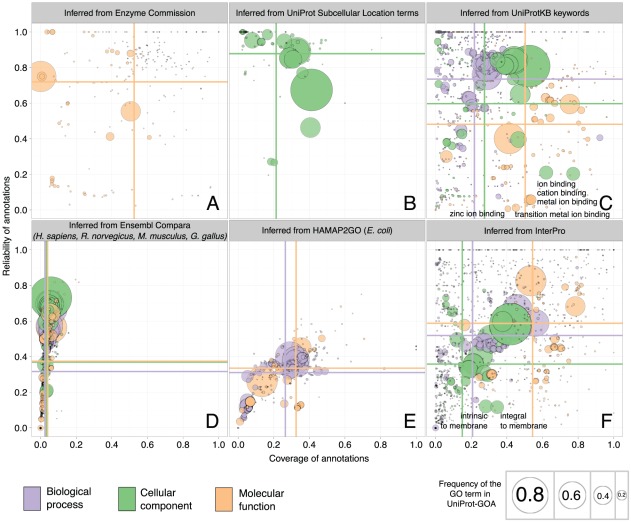
The quality of the 16-01-2008 UniProt-GOA release, evaluated by the 11-01-2011 UniProt-GOA release. Each reference code is evaluated separately: (A) Inferred from Enzyme Commission, (B) Inferred from UniProt Subcellular Location terms, (C) Inferred from UniProtKB keywords, (D) Inferred from Ensembl Compara, (E) Inferred from HAMAP2GO, and (F) Inferred from InterPro. The 12 model organisms included in the analysis are *Homo sapiens, Mus musculus, Rattus norvegicus, Caenorhabditis elegans, Drosophila melanogaster, Arabidopsis thaliana, Gallus gallus, Danio rerio, Dictyostelium discoideum, Saccharomyces cerevisiae, Schizosaccharomyces pombe*, and *Escherichia coli* K-12. The ontology is denoted by the color of the disc, while the area of the disc reflects the frequency of the GO term in the 16-01-2008 UniProt-GOA release. The colored lines correspond to the mean values for the respective axes. To be visualized in this plot, a GO term needs to have assigned at least 10 electronic annotations in the 16-01-2008 UniProt-GOA release and at least 10 experimental annotations in the 11-01-2011 UniProt-GOA release.

To investigate differences in quality among the various sources of electronic annotations in UniProt-GOA, we repeated our analysis for each of them. The six sources can be classified in two main categories: mapping of keywords from other databases (UniProtKB keywords, UniProt Subcellular Location terms, InterPro, and Enzyme Commission) and the use of comparative genomics in functional annotation (Ensembl Compara for eukaryotes and HAMAP2GO for microbial genomes) (Fig. 6).

Two sources of electronic annotations are restricted to single ontologies: the Enzyme Commission (EC) numbers map to MF GO terms, and subcellular location terms of the UniProt database map to CC GO terms (Fig. 6 A/B). Both annotation sources are applied to a comparatively small number of terms, but their reliability is remarkably high: on this restricted set of GO terms, they outperform other sources of electronic annotation (Fig. 6, Fig. S2 in Text S1, and Fig. S3 in Text S1).

The bulk of electronic annotations are inferred from the UniProt and InterPro databases (Fig. S4 in Text S1). With UniProtKB keywords, GO annotations are inferred using a correspondence table between Swiss-Prot keywords associated with UniProt entries and GO terms. Note that UniProt entries consist of a small minority of manually annotated entries (“Swiss-Prot entries”) and a large body of entries (“TrEMBL entries”) automatically annotated by a rule-based system (“UniRules”). With InterPro, GO annotations are inferred from a correspondence table between InterPro sequence and structure signatures and GO terms. Despite similarities in the two approaches, UniProt-based annotations show considerably higher average reliability than their InterPro-based counterparts (Fig. 6 C/F, horizontal lines). In terms of average coverage, the two approaches show similar performance (Fig. 6 C/F, vertical lines).

Substantial manual curation is involved in obtaining electronic annotations from the two sources that rely on comparative genomics: Ensembl Compara electronic annotations transfer experimental annotations among inferred one-to-one orthologs in a subset of model organisms, and HAMAP2GO electronic annotations rely on manually created rules to propagate experimental annotations within a family of microbial proteins. Despite the intricacies involved in the annotation pipeline, these two sources have the lowest mean coverage and reliability among the six analyzed sources (Fig. 6 D/E). However, note that the HAMAP rules have taxonomic restrictions on propagation that are not included in the HAMAP2G0 pipeline. Hence, some aspects of HAMAP are not captured in UniProt-GOA, and therefore are not analyzed here.

This overall low reliability—a consequence of many rejected annotations—indicates that GOA strategies based on comparative genomics are currently less reliable than approaches based on sequence features (UniProtKB keywords and InterPro).

### Quality of electronic annotations and the number of assigned GO terms are different among the model organisms

**Figure 7:**
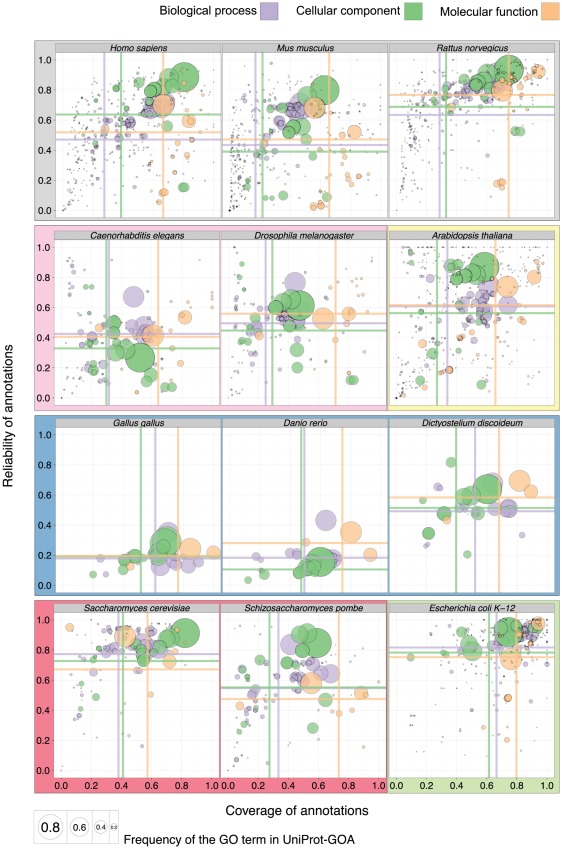
Quality of the 16-01-2008 UniProt-GOA release, evaluated by the 11-01-2011 UniProt-GOA release; each model organism is evaluated separately. Common background shading denotes a depiction of the same set of GO terms (full data is presented in Fig. S8 in Text S1). The ontology is denoted by the color of the disc, while the area of the disc reflects the frequency of the GO term in the 16-01-2008 UniProt-GOA release. To be visualized in this plot, a GO term needs to have assigned at least 10 electronic annotations in the 16-01-2008 UniProt-GOA release and at least 10 experimental annotations in the 11-01-2011 UniProt-GOA release for each model organism. The colored lines correspond to the mean values for the respective axes.

To investigate the difference in electronic annotation quality among the model organisms, we repeated our analysis for each model organism separately. Overall, repeating the analysis confirmed our general findings above. However, we observed variations among organisms, both in the number of available annotations and their quality (Fig. 7, Fig. S5 in Text S1, Fig. S6 in Text S1, and Fig. S7 in Text S1).

Organisms with the largest number of changes—confirmations and rejections—tend to have the highest quality of annotation: the three unicellular organisms and the three mammals (Fig. 6, top and bottom rows, Fig. S7 in Text S1). Experimenting, describing and interpreting results on unicellular organisms is arguably more straightforward than on multicellular organisms; it might explain the relatively high quality of electronic annotations for the three unicellular model organisms (Fig. 7, bottom row). The average quality measures for the three mammals—*Homo sapiens*, *Mus musculus*, and *Rattus norvegicus*—are comparably high (Fig. 7, top row), but many specific low-quality annotations somewhat reduce the means of reliability and coverage.

Our observation that general GO terms tend to have higher reliability holds for each model organism. Nevertheless, assigning mainly general GO terms guarantees neither high reliability nor high coverage. We observe the worst electronic annotation quality on *Gallus gallus*, *Danio rerio* and *Dictiostelium discoideum* gene products, despite a mean specificity of 1.79, versus 4.47 for mammals.

### The reliability of electronic annotations rivals that of non-experimental curated annotations

**Figure 8:**
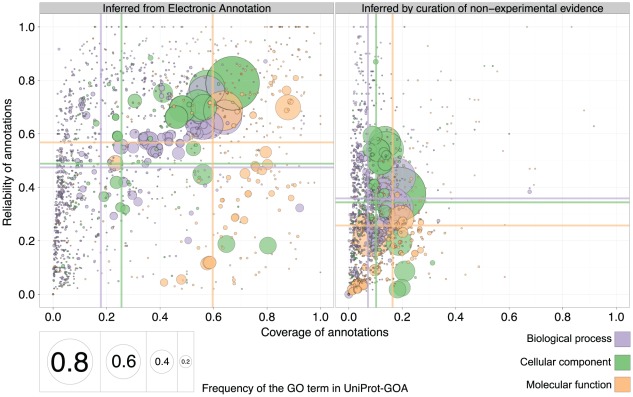
Quality of electronic and curated annotations on a common set of GO terms. Quality of the 16-01-2008 UniProt-GOA release is evaluated by the 11-01-2011 UniProt-GOA release; coverage is on the x-axis and reliability is on the y-axis. The ontology is denoted by the color of the disc, while the area of the disc reflects the frequency of the GO term in the 16-01-2008 UniProt-GOA release. The colored lines correspond to the mean values for the respective axes. To be visualized in the plot, a GO term needs to have assigned at least 10 electronic/curated annotations in the 16-01-2008 UniProt-GOA release, and at least 10 experimental annotations in the 11-01-2011 UniProt-GOA release.

To put the quality of electronic annotations in perspective, we contrasted them to curated annotations (evidence codes RCA, ISS, TAS, NAS, and IC), i.e. annotations inferred by curators without direct experimental evidence (Fig. 8). Curated annotations contain annotations assigned using evidence codes perceived as of particularly high quality: for instance, del Pozo et al. [5] consider the TAS evidence code to “offer the highest confidence [along with the IDA evidence code]”. Buza et al. [6] rank TAS and IC evidence code second only to the group of annotation codes that rely on direct experimental evidence. In Benabderrahmane et al. [7], TAS is the only evidence code to receive the weight of 1.0.

Compared to electronic annotations, it is not surprising that curated annotations have a considerably lower average coverage (Fig. 8, vertical lines). Indeed, the main appeal of electronic annotations is precisely that they scale efficiently to large quantities of data. But in terms of reliability, and contrary to current beliefs, curated annotations that use evidence other than experiments from primary literature do not fare better than electronic annotations (Fig. 8, horizontal lines, Fig. S9 in Text S1). In fact, we observed a higher reliability for electronic annotations than for curated annotations (0.52 vs. 0.33).

A more detailed analysis revealed that the lower mean reliability of curated annotations in the 16-01-2008 UniProt-GOA release is mainly due to removed annotations with evidence code Reviewed Computational Analysis (RCA) (Fig. S10 in Text S1). The low reliability of RCA annotations is caused by the removal of many RCA annotations assigned to the *M. musculus* gene products (Fig. S7 in Text S1, yellow bar in the panel denoted *Mus musculus*); these were removed as there were concerns about the veracity of results from some papers that had been annotated (Emily Dimmer, personal correspondence).

When we exclude annotations assigned using the RCA evidence code, the reliability of non-experimental curated annotations rises to 0.58. But even then, the reliability of electronic annotations (0.52) remains competitive with that of curated annotations (Fig. S11 in Text S1).

## Discussion

Electronic annotations constitute the bulk of GO annotations, yet their correctness has not been systematically assessed until now. Direct, experimental verification by means of new experiments would be prohibitively expensive even for a small subset of the annotations. Instead, we sought to exploit existing, but newly available experimental data to evaluate electronic annotations. Specifically, we defined and used a measure we call *reliability* as an indicator of correctness: a GO term has high reliability if, in a subsequent release, many associated electronic annotations are confirmed experimentally while few associated annotations are removed or explicitly negated. This approach at verifying electronic annotations is both efficient (as it reuses existing experiments) and powerful (as it potentially applies to any term). At the same time, the measure is only as accurate and representative as the newly recorded experimental annotations. For instance, there are far more “positive” function annotations than “negative” ones (annotations with a “NOT” qualifier, which indicates lack of function), which could result in inflated reliability estimates. On the other hand, we attempt to compensate for this bias by considering all removed electronic annotations as negative ones. While it might be argued that the removal of an electronic annotation does not necessarily imply that it is wrong, from a user standpoint, the removal of an annotation hardly suggests that it can be relied upon.

Despite analyzing 193,027 gene products, our approach leaves out a number of uninformative electronic annotations, which are neither confirmed nor rejected in a given time interval. Due to the incomplete nature of GO (sometimes referred to as the “open-world” assumption), absence of an annotation does *not* imply absence of the corresponding function. This is reflected by the fact that most gene products in GOA have been updated at least once—with the period between updates lasting as long as 12 years (Fig. S12 in Text S1).

Electronic annotations have often been perceived as unreliable, but our study provides a more differentiated picture. First, we observed that the reliability and, to a lesser extent, the specificity of electronic GO annotation has steadily improved in recent years. This is a remarkable achievement, given that the number of electronic annotations has been growing exponentially during the same time period [2].

Second, despite these overall encouraging results, there are significant variations in performance among the different types of electronic annotations. The two most reliable sources also happen to be the most specialized ones: annotations derived from UniProt Subcellular Location terms and EC numbers. This suggests that specialization can be advantageous.

Also highly reliable are annotations obtained from mapping Swiss-Prot keywords associated with UniProtKB entries to GO terms. In particular, the high mean reliability of predictions of Biological Process GO terms stands out, on what is arguably the most difficult ontology to assign [14]. There are nevertheless a handful of general UniProtKB keywords derived GO terms that have low reliability (Fig. 6); in particular, Molecular Function terms related to metal ion binding have proven to be unreliable throughout all three analyzed UniProt-GOA releases due to a number of removed annotations (GO terms denoted in Fig. 6 C, Dataset S1; an interactive plot is available at http://people.inf.ethz.ch/skuncan/SupplementaryVisualization2.html). In addition, a few annotations related to ion transport were explicitly rejected with the ‘NOT’ qualifier, e.g. UniProtID Q6R3K9 now has a ‘NOT’ annotation for “iron ion transport”, UniProtID Q3YL57 now has a ‘NOT’ annotation for “sodium ion transport”, and UniProtID Q9UN42 now has a ‘NOT’ annotation for “monovalent inorganic cation transport”.

Since the UniProt database includes manually annotated entries (“Swiss-Prot entries”) in addition to electronically annotated (“TrEMBL entries”), this could introduce some circularity in our analysis. However, the proportion of manually annotated entries in UniProt is very small (3.06% in the September 2011 UniProt release), so any bias so incurred cannot affect our conclusions. The importance of the automated component of the UniProt pipeline is also reflected in the large number of electronic annotations derived from it—almost a quarter of all electronic annotations (Fig. S4 in Text S1).

Besides UniProtKB keywords, InterPro sequence and structure signatures constitute the other large source of electronic annotations (42%; Fig. S4 in Text S1). Their average reliability is however not as good as UniProtKB keywords-derived terms. Consider for instance the Cellular Component term “integral to membrane” and its parent term “intrinsic to membrane” (Fig. 6F). The reliability of annotations associated with these terms was low across several releases (http://people.inf.ethz.ch/skuncan/SupplementaryVisualization3.html). These observations are consistent with a recent article reporting “promiscuous hits limited to solely [signal peptide or transmembrane helix] part among clearly unrelated proteins” [15]. Moreover, we observed more InterPro annotations rejected with the ‘NOT’ qualifier than UniProtKB-based annotations (Dataset S1). For example, UniProtIDs Q8IZE3, Q96RU7, and Q8BKG3 now have a ‘NOT’ annotation for “kinase activity”; UniProtID Q2L385 now has a ‘NOT’ annotation for “channel activity”; UniProtIDs Q9LQ10, Q8GYY0, and Q06429 now have a ‘NOT’ annotation for “1-aminocyclopropane-1-carboxylate synthase activity.”

As for strategies based on comparative genomics, namely HAMAP2GO and Ensembl Compara, they yielded the least reliable annotations of those we analyzed. But because they have been introduced in the UniProt-GOA releases relatively recently, we could only assess their performance on one or two overlapping time intervals (Fig. S13 in Text S1). If transient, the low reliability of an annotation source could be the result of a large change in the annotation pipeline that ultimately results in more reliable resource. For instance, when looking for the cause of low reliability for the annotations Inferred from HAMAP2GO (Fig. 6 E), we found the HAMAP2GO file—mapping HAMAP annotations to GO terms—is currently being substantially revised (Alan Bridge and Emily Dimmer, personal correspondence). A recent change in policy towards more conservative predictions resulted in the large number of removed annotations we observed. Because of the lagging nature of our quality measures, we will only be able to assess the new pipeline in a few releases' time.

Despite these considerable variations among sources of annotations, all electronic annotations are currently labeled with the same evidence code (“IEA”)—with the source information relegated to the more obscure “which/from” attribute. As many users and tools tend to ignore the latter database column, we recommend making these differences more explicit by introducing multiple evidence codes for electronic annotations; the new evidence codes might take into account the subdivisions available in the ECO ontology (http://obofoundry.org/cgi-bin/detail.cgi?id=evidence_code).

The third and arguably most unexpected finding of this study is that the reliability of electronic annotations rivals that of annotations assigned by an expert curator using sources other than direct experimental evidence (Fig. 8, horizontal lines). At the same time, the coverage of electronic annotations—which measures the ability to predict future experimental annotations—is far superior (Fig. 8, vertical lines). For example, the mean reliability of the BP ontology is slightly lower when inferred from electronic annotations than when the annotations are based on sequence similarity and approved by the curator (evidence code ISS). Still, the mean reliabilities for the CC and MF ontologies are slightly higher for electronic annotations, and the mean coverage of electronic annotations for all three ontologies is visibly higher (Fig. S14 in Text S1).

This challenges the widespread notion that annotations inferred by algorithms are less reliable than annotations inferred by curators using evidence other than direct experimental evidence found in primary literature—a notion that might have had validity when automated annotations consisted of relatively crude approaches, such as global sequence similarity with ready-made thresholds. Although occasionally still in use, such annotation strategies have been largely superseded by the approaches highlighted here and described elsewhere in more detail [9], [16], [17].

### Conclusion

To narrow the gap between the number of sequenced gene products and those with functional annotation, computational methods are indispensable [18], [19], even more so for the non-model organisms (Fig. S4 in Text S1). We introduced three measures to evaluate the quality of electronic annotations: one accounts for the *specificity* of the assigned GO term, and two—*reliability* and *coverage*—assess the performance of electronic annotation sources by tracking changes in subsequent releases of annotation files.

Although the performance of electronic annotations varies among inference methods (“sources”), the overall quality of electronic annotations rivals the quality of curated non-experimental annotations.

This is not to say that the curators have made themselves redundant. On the contrary, as we highlight above, most electronic annotations heavily rely on manually curated UniProtKB keywords and InterPro entries. Moreover, given the essential role of curators in embedding experimental results into ontologies, so does the present study.

## Materials and Methods

### Data

We used the January 2011 release of the OBO-XML file to obtain the GO terms, definitions and the ontology structure needed in the analysis. The file was downloaded from the GO FTP site http://archive.geneontology.org/latest-full/.

The annotations (mappings of gene products to GO terms) were downloaded from the European Institute for Bioinformatics (EBI) FTP site ftp://ftp.ebi.ac.uk/pub/databases/GO/goa/UNIPROT/. Each file, created as part of the UniProt Gene Ontology Annotation (UniProt-GOA) project [9], is a many-to-many mapping of UniProtKB IDs to GO terms. All dates mentioned in this study refer to the release date of these annotation files, not the date attribute of individual annotations.

We analyzed 193,027 UniProtKB IDs; GO terms can be assigned to these sequences using any of the evidence or reference codes. The distribution of annotations among the 12 Gene Ontology Reference genomes [10] is shown in Fig. S6 in Text S1. This set of model organisms has by far the largest number of high-quality experimental annotations, allowing us to make the most reliable estimate of the annotation quality (Fig. S1 in Text S1).

The structure of the GO vocabulary is changing as a response to consistency checks, new biological insights, and intricacies involved in annotating various model organisms [20]–[22]. To account for these changes, for each pair of GO releases analyzed we only consider terms that are present in both releases.

### Gene Ontology meta-information

The source of an annotation is recorded in the evidence code (http://www.geneontology.org/GO.evidence.shtml). We group GO evidence codes into 3 broad categories: 1) codes reflecting annotations assigned by curators using direct experimental evidence from the literature (*experimental* evidence codes EXP, IMP, IGI, IPI, IEP, IDA), 2) codes reflecting annotations inferred by curators using other types of evidence (*curated* evidence codes ISS, RCA, IC, NAS, TAS) and 3) *electronic* evidence code (IEA), denoting annotations which are inferred computationally (Fig. 1). Several evidence codes were not included in the analysis: they are either used to indicate curation status/progress (ND), are obsolete (NR), or there is not enough data to make a reliable estimate of their quality (ISO, ISA, ISM, IGC, IBA, IBD, IKR, IRD).

A reference code captures the source of an electronic annotation. We analyze six reference codes available in UniProt-GOA: three are based on cross-referencing keywords from other databases: UniProtKB keywords, UniProt Subcellular Location terms, and Enzyme Commission [23], [24]; two are based on the propagation of annotations within a family of proteins: InterPro and HAMAP2GO [25], [26]; one reference code uses comparative genomics in projecting experimental annotations to unannotated inferred one-to-one orthologs—Ensembl Compara [27].

When a ‘NOT’ qualifier accompanies an annotation, it explicitly states that the gene product is not associated with the respective GO term. A subtle use of the ‘NOT’ qualifier comes into play because the isoform distinctions are not reflected in the annotation files at this time; a gene product can be mapped to the GO term in a given spatial/temporal context, but the mapping is *not* valid in another context (Judith Blake and Pascale Gaudet, personal correspondence). Such gene products will be mapped to one GO term twice—one accompanied by a ‘NOT’ qualifier and one without it. For consistency, we ignore all such occurrences. The 11-01-2011 UniProt-GOA release contains 493 gene products with such annotations.

### Qualitative evaluation of Gene Ontology annotations using successive releases of the UniProt-GOA file

All analyses are performed on overlapping 3-year periods between 2006 and 2011. Unless stated otherwise, we show the results associated with the most recent period (2008–2011).

The three measures of quality we introduced are specificity, reliability, and coverage. For clarity, the definitions are given and described for electronic annotations. Nevertheless, any subset of annotations can be analyzed this way, e.g. annotations assigned using one or a subset of evidence or reference codes.

We measure the *specificity* (opposite of generality) of a GO term GO_i_ with respect to its information content [10], [28], [29]:
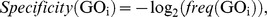
where freq(GO_i_) is the frequency of GO_i_ among all annotations considered.

To calculate the *reliability* for a GO term, we count all the *confirmed* and *rejected* electronic annotations associated with this term (Fig. 2 A). An electronic annotation is confirmed if it is corroborated by a new (added during the time interval) experimental annotation. An electronic annotation is rejected if it is falsified by a new experimental annotation that comes with a ‘NOT’ qualifier, or if this electronic annotation has been removed in the later UniProt-GOA release. More formally,
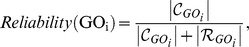
where 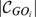 is the set of confirmed annotations associated with term GO_i_ and 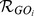 is the set of rejected and removed annotations associated with term GO_i_.

To calculate the *coverage* for a GO term in a UniProt-GOA release, we count all the new experimental annotations in the later UniProt-GOA release correctly *predicted* by an electronic annotation in the earlier release, and those not correctly predicted (missed) by electronic annotations in the earlier release (Fig. 2 B). More formally,
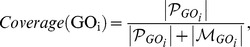
where 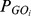 is the set of correctly predicted new experimental annotations associated with term GO_i_ and 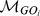 is the set of “missed” new experimental annotations associated with term GO_i_.

To calculate any of the measures of quality, we take into account the GO Direct Acyclic Graph (DAG) structure. To calculate the frequency of a GO term, we account for all annotations derived by inheritance. Consequently, the specificity of any child term is necessarily greater than or equal to the specificity of its parents. When calculating reliability, an annotation that is replaced by a more specific annotation (a descendent) is not considered rejected, as the descendent still implies it. Similarly, an annotation is confirmed by the arrival of an experimentally ascertained descendent, as the more specific term implies the more general term. Conversely, if an annotation is followed by the arrival of a less specific experimental annotation, only the subset of its ancestral terms implied by the less specific experimental annotation is deemed as confirmed; the rest is uninformative (neither confirmed, rejected, or removed).

All the results of the described analysis are available as Dataset S2.

### Visualization

The analysis was done using a combination of in-house Java classes, SQL queries to the custom database, and R scripts. Summaries were done using the plyr package of the R language [30]; all plots were created using the ggplot2 package of the R language [31], and the interactive plots were created using the googleVis package of the R language; the respective R packages are available from the CRAN repository. REVIGO web server [32] was used to summarize the lists of GO terms and select those highlighted in the Results section.

## Supporting Information

Text S1 Supplementary figures.
(PDF)

Dataset S1 A zip archive containing a list of removed and rejected annotations; each table contains the data for one evidence or reference code.
(ZIP)

Dataset S2 A list of GO terms and their corresponding Reliability, Coverage, and Generality for each model organisms and for each analyzed reference or evidence code in the three analyzed intervals.
(ZIP)
