# Supplementary material for: Quality of Computationally Inferred Gene Ontology Annotations
Source: PLoS Comput Biol. 2012 May 31;8(5):e1002533. doi: 10.1371/journal.pcbi.1002533 (PMC3364937; doi:10.1371/journal.pcbi.1002533)
Supplement: Text S1 — Supplementary figures. (PDF) [file pcbi.1002533.s001.pdf]

Quality of Computationally Inferred Gene Ontology Annotations
Skunca N, Altenhoff AM, Dessimoz C, PLoS Comput Biol 2012
DOI: 10.1371/journal.pcbi.1002533

Supplementary Figures

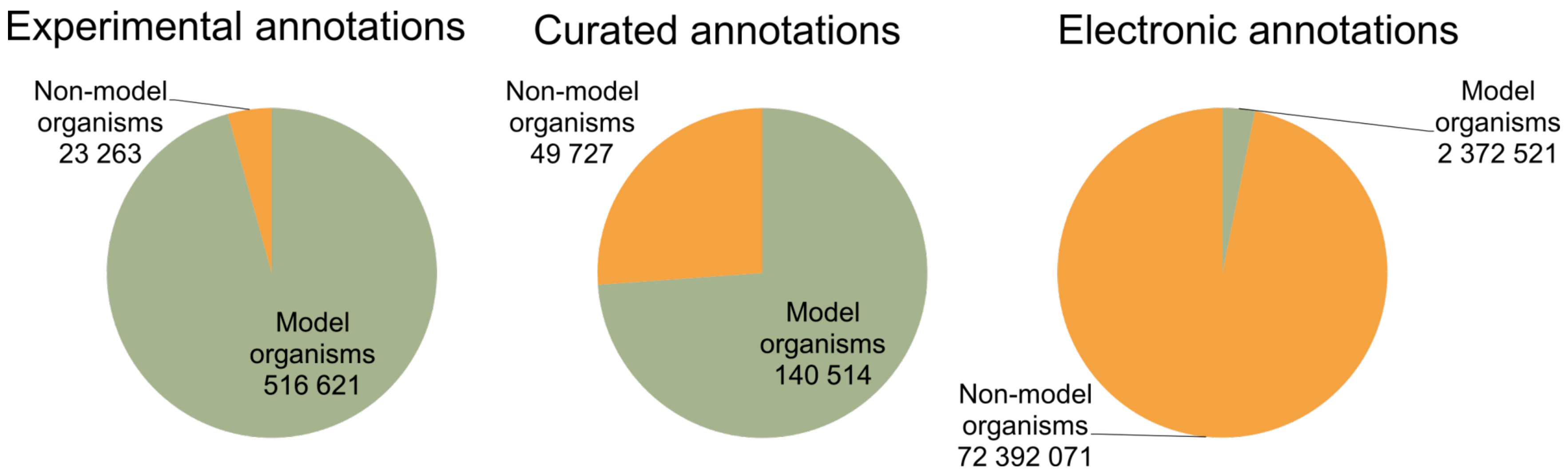

Figure S1. Distribution of experimental, curated, and electronic annotations among the 12 model and 241 860 non-model organisms in the 11-01-2011 UniProt-GOA release. The numbers account for annotations available in the UniProt-GOA release, i.e. we do not report the annotations available through the definition of the ontology. The 12 model organisms are *Homo sapiens, Mus musculus, Rattus norvegicus, Caenorhabditis elegans, Drosophila melanogaster, Arabidopsis thaliana, Gallus gallus, Danio rerio, Dictyostelium discoideum, Saccharomyces cerevisiae, Schizosaccharomyces pombe*, and *Escherichia coli* K-12. Out of non-model organisms, 838 have at least one experimental annotation, while the rest have no experimental annotations.

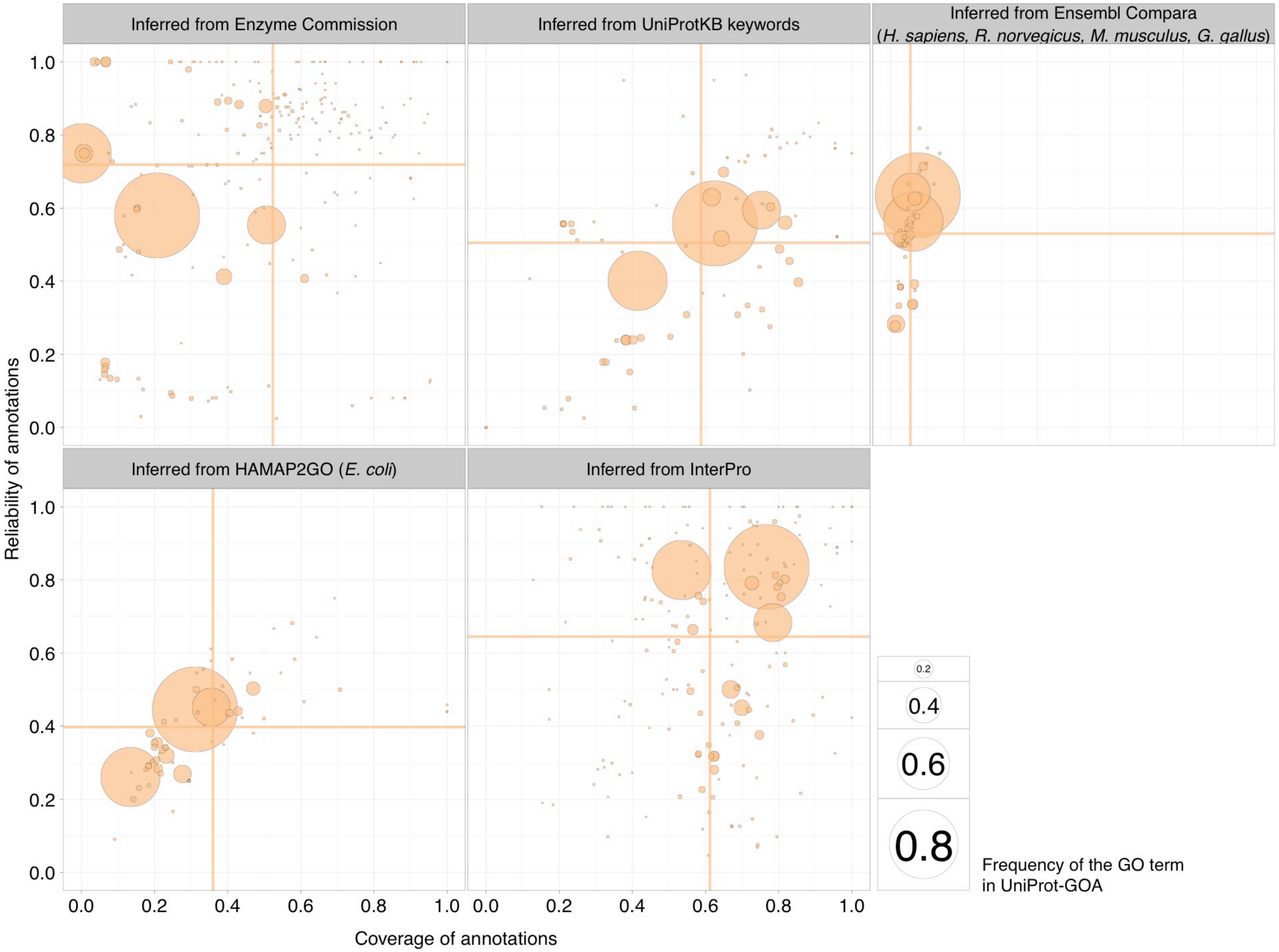

Figure S2. The quality of the 16-01-2008 UniProt-GOA release, evaluated using the 11-01-2011 UniProt-GOA release; comparison on the subset of molecular function GO terms used in electronic annotations inferred from Enzyme Commission mappings.

The 12 model organisms are *Homo sapiens, Mus musculus, Rattus norvegicus, Caenorhabditis elegans, Drosophila melanogaster, Arabidopsis thaliana, Gallus gallus, Danio rerio, Dictyostelium discoideum, Saccharomyces cerevisiae, Schizosaccharomyces pombe*, and *Escherichia coli* K-12.

The area of the disc reflects the frequency of the GO term in the 16-01-2008 **UniProt**-GOA release. To be visualized in this plot, a GO term needs to have assigned at least 10 electronic annotations in the 16-01-2008 **UniProt**-GOA release and at least 10 experimental annotations in the 11-01-2011 **UniProt**-GOA release. The orange lines correspond to the mean values for the respective axes.

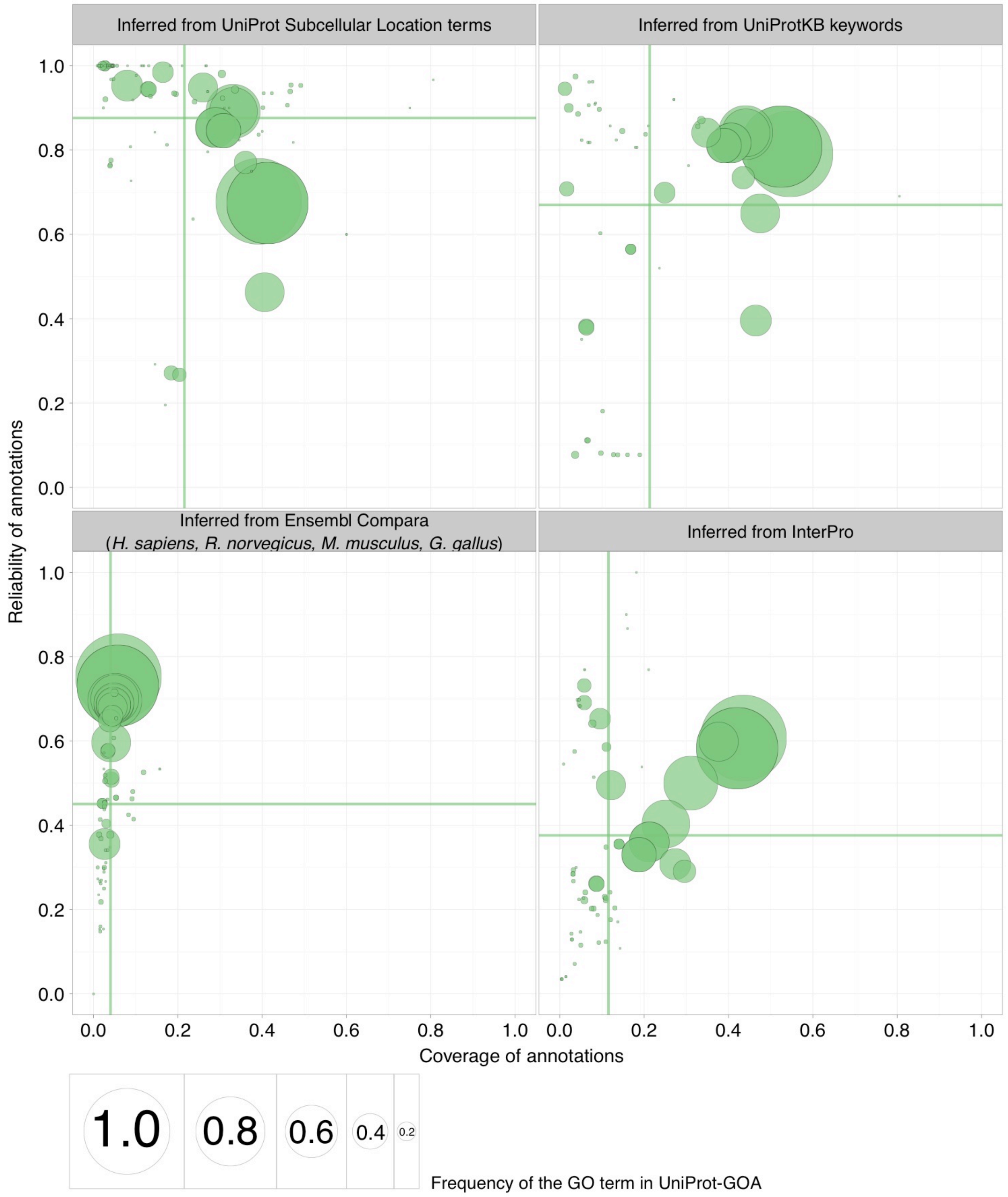

Figure S3. The quality of the 16-01-2008 UniProt-GOA release, evaluated using the 11-01-2011 UniProt-GOA release; comparison on the subset of the cellular component GO terms used in electronic annotations inferred from UniProt subcellular mappings. The 12 model organisms are *Homo sapiens, Mus musculus, Rattus norvegicus, Caenorhabditis elegans, Drosophila melanogaster, Arabidopsis thaliana, Gallus gallus, Danio rerio, Dictyostelium discoideum, Saccharomyces cerevisiae, Schizosaccharomyces pombe*, and *Escherichia coli* K-12. The area of the disc reflects the frequency of the GO term in the 16-01-2008 **UniProt**-GOA release. To be visualized in this plot, a GO term needs to have assigned at least 10 electronic annotations in the 16-01-2008 **UniProt**-GOA release and at least 10 experimental annotations in the 11-01-2011 **UniProt**-GOA release. The green lines correspond to the mean values for the respective axes.

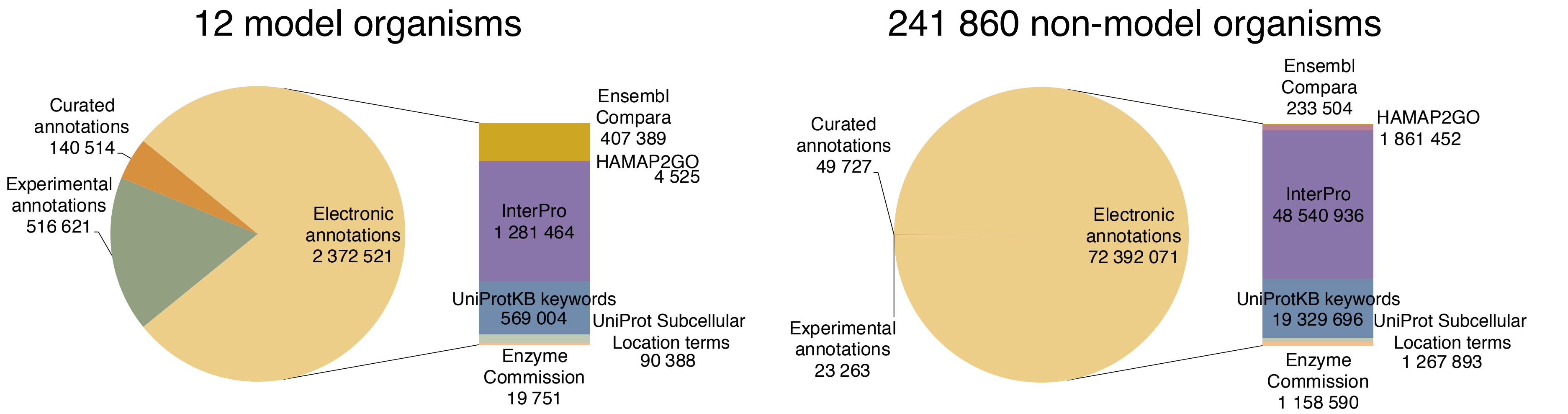

Figure S4. Distribution of experimental and electronic annotations among the 12 model and 241 860 non-model organisms in the 11-01-2011 GOA release. The numbers account for annotations available in the GOA release, i.e. we do not report the annotations available through the definition of the ontology. The 12 model organisms are *Homo sapiens, Mus musculus, Rattus norvegicus, Caenorhabditis elegans, Drosophila melanogaster, Arabidopsis thaliana, Gallus gallus, Danio rerio, Dictyostelium discoideum, Saccharomyces cerevisiae, Schizosaccharomyces pombe*, and *Escherichia coli* K-12.

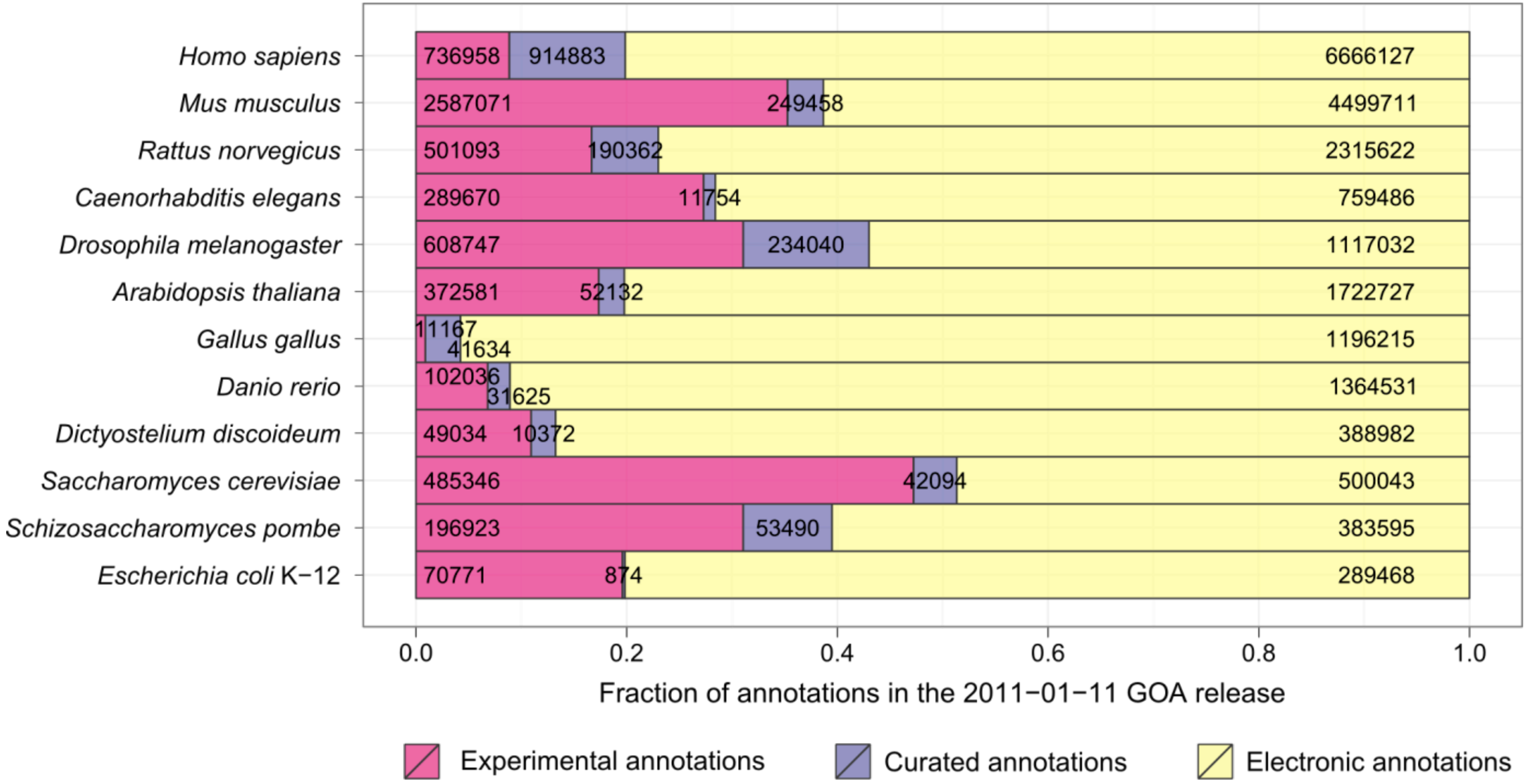

Figure S5. Distribution of annotations from the three sources—experimental, curated and electronic—among the 12 reference genomes. Numbers denote the annotations available for the respective bar; these include annotations available in the 11-01-2011 GOA release, as well as annotations available through the definition of the ontology (i.e. all parent GO terms).

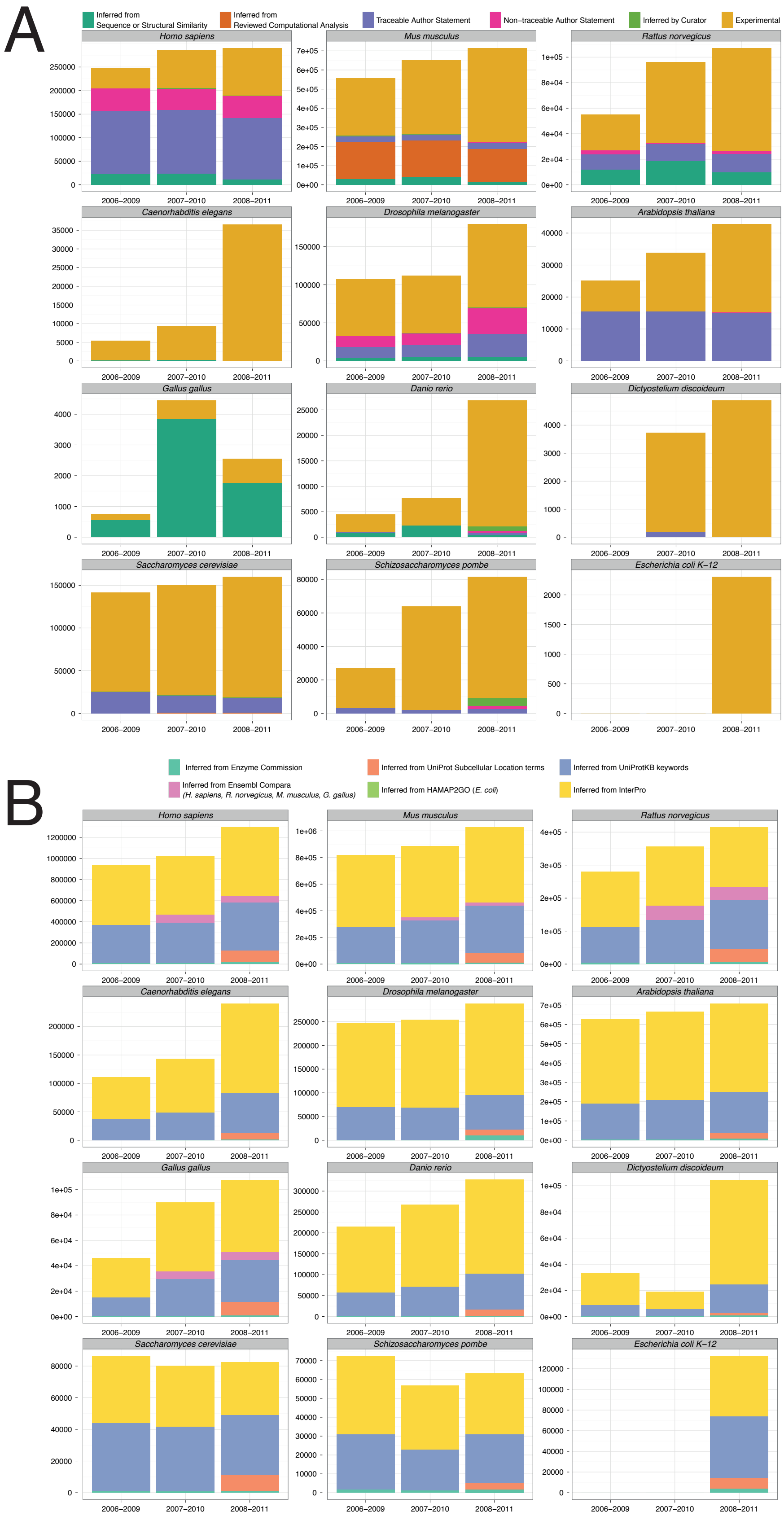

Figure S6. Curated (A) and electronic (B) annotations we analyze for the 12 model organisms. Each panel summarizes the data for the model organism denoted in the header; the color denotes the evidence or the reference code, and the y axis denotes the number of annotations analyzed for the period denoted on the x axis.

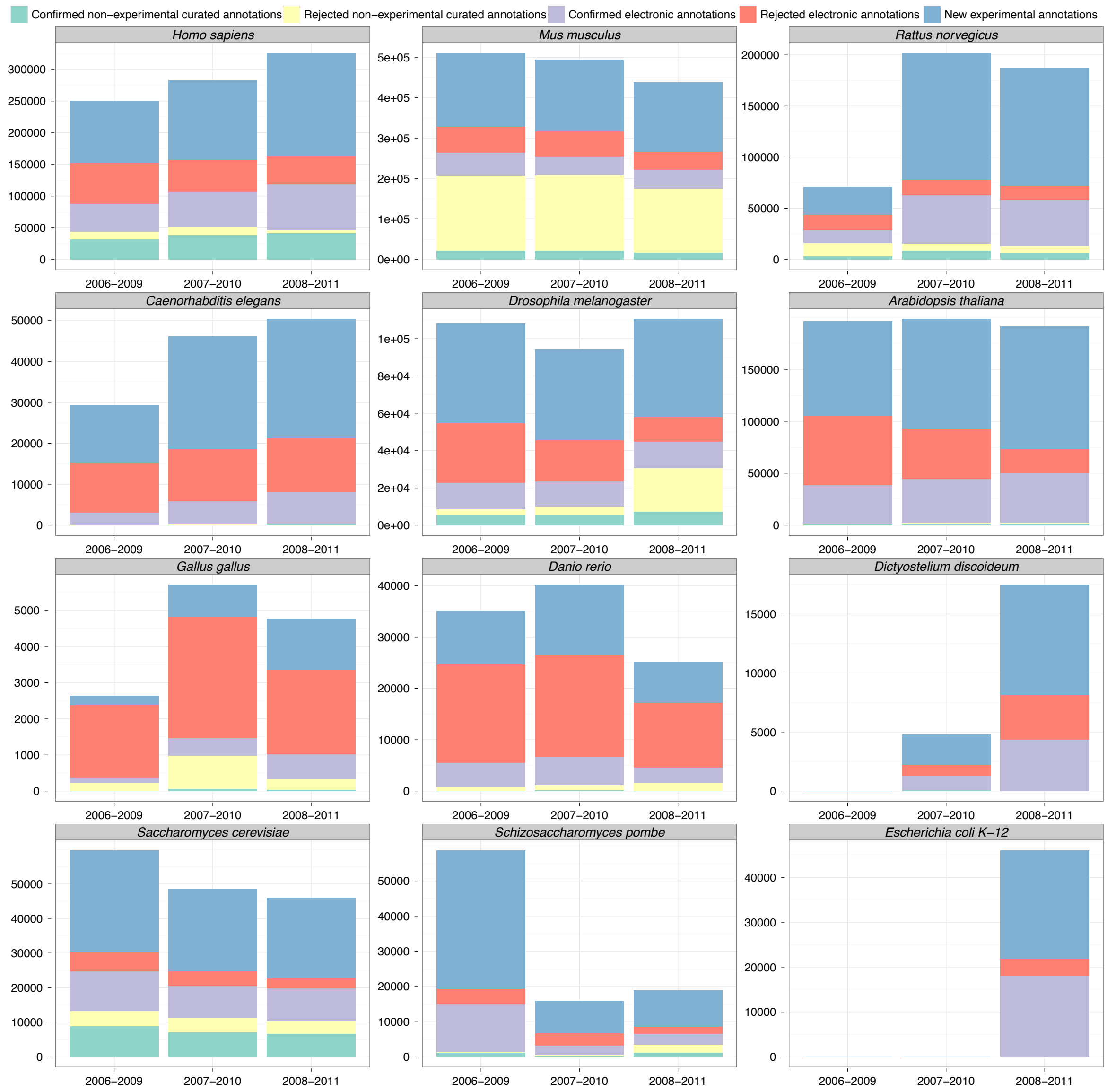

Figure S7. Counts of confirmed, rejected and newly available experimental annotations we analyze for the 12 model organisms. Each panel summarizes the data for the model organism denoted in the header; the color denotes the group of evidence codes and the y axis denotes the number of annotations counted in the period denoted on the x axis.

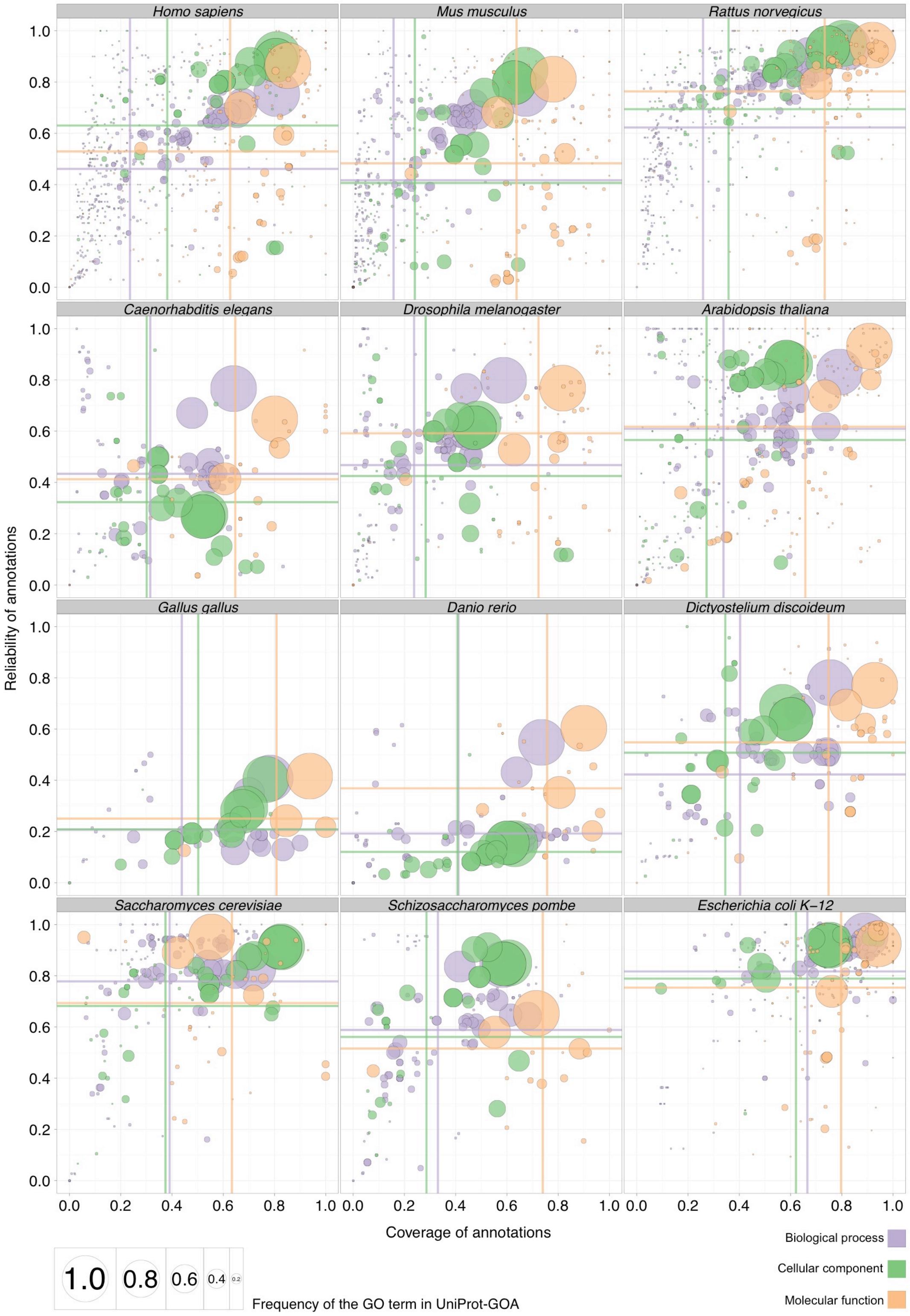

Figure S8. The quality of the 16-01-2008 GOA release, evaluated by the 11-01-2011 GOA release; each model organism is evaluated separately. The ontology is denoted by the color of the disc, while the area of the disc reflects the frequency of the GO term in the 16-01-2008 GOA release. To be visualized in this plot, a GO term needs to have assigned at least 10 electronic annotations in the 16-01-2008 GOA release and at least 10 experimental annotations in the 11-01-2011 GOA release. The colored lines correspond to the mean values for the respective axes.

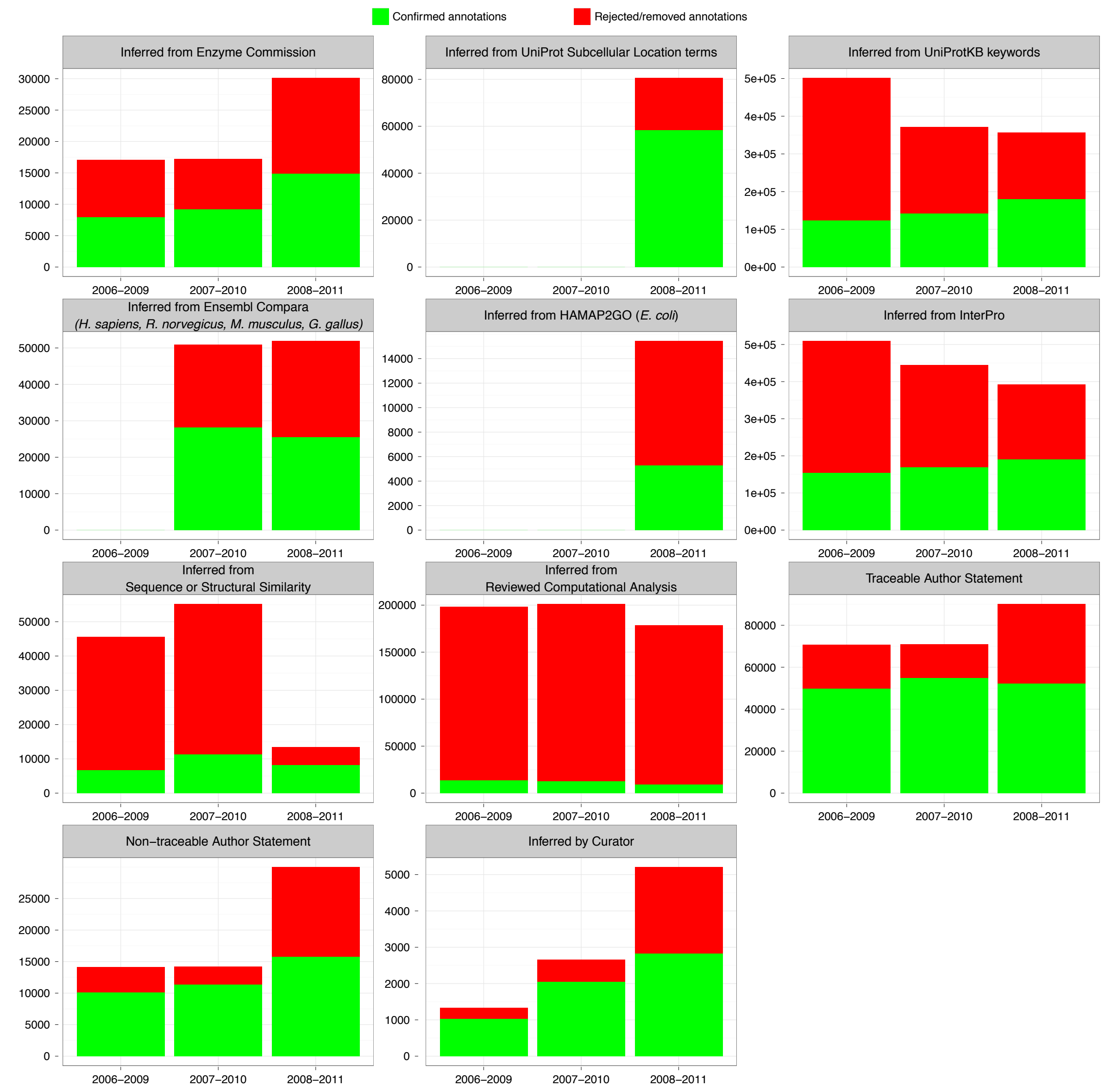

Figure S9. Counts of confirmed and rejected annotations for the analyzed evidence or reference codes. The y axis denotes the number of confirmed (green) or rejected/removed (red) annotations counted in the period denoted on the x axis.

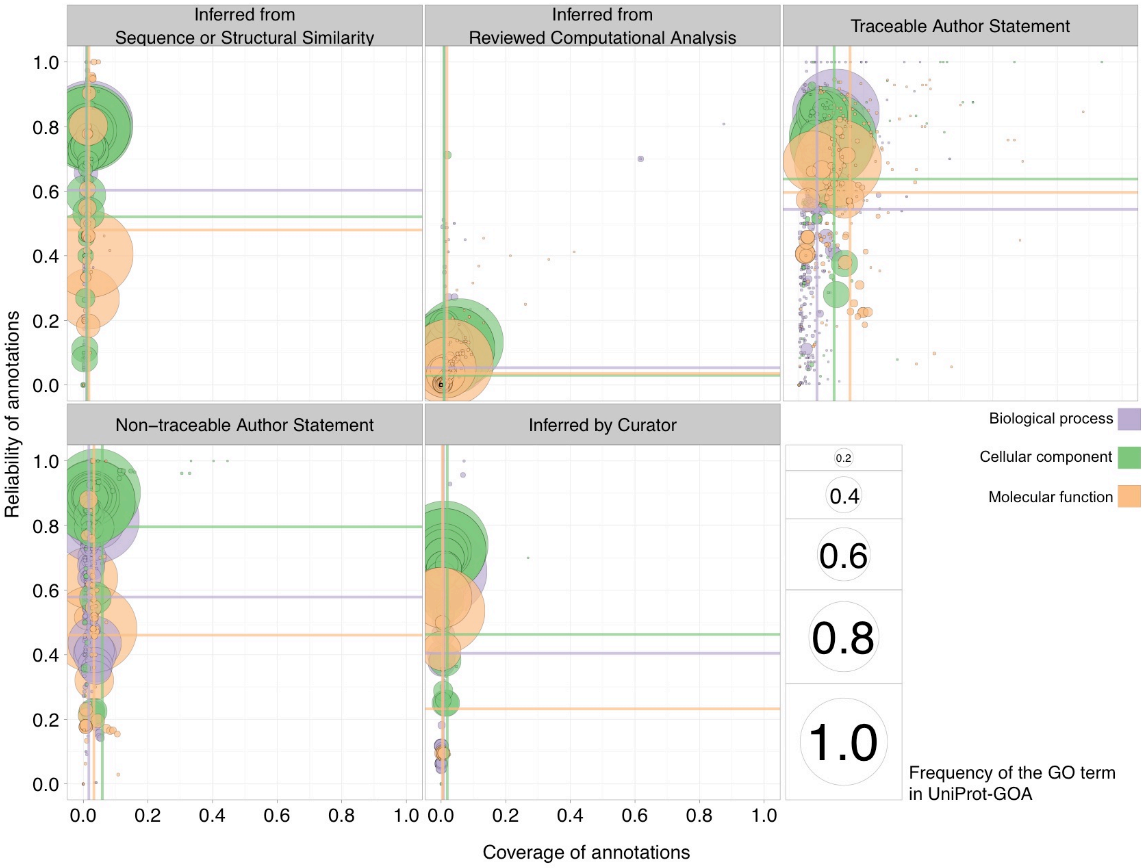

Figure S10. The quality of the 16-01-2008 GOA release, evaluated by the 11-01-2011 GOA release; each evidence code is evaluated separately. The ontology is denoted by the color of the disc, while the area of the disc reflects the frequency of the GO term in the 16-01-2008 GOA release. The colored lines correspond to the mean values for the respective axes. To be visualized in this plot, a GO term needs to have assigned at least 10 electronic annotations in the 16-01-2008 GOA release and at least 10 experimental annotations in the 11-01-2011 GOA release.

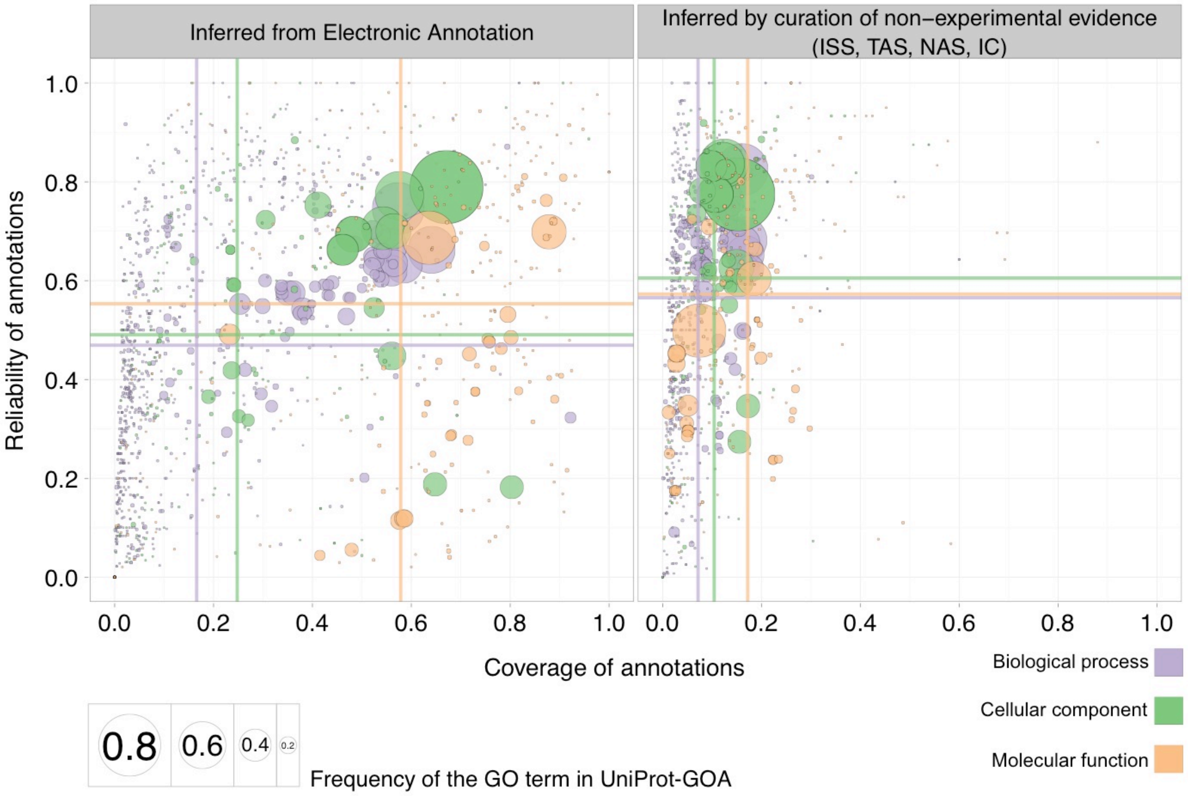

Figure S11. Quality of electronic and curated annotations on a common set of GO terms; we exclude annotations assigned with Reviewed Computational Analysis (RCA) evidence code. *Electronic* sources are inferred from InterPro, UniProt, UniProt (subcellular), Enzyme Commission, Ensembl Compara, and HAMAP; *curated* sources are Inferred by Sequence Similarity (ISS), Traceable Author Statement (TAS), Non-traceable Author Statement (NAS), and Inferred by Curator (IC). Quality of the 16-01-2008 GOA release is evaluated by the 11-01-2011 GOA release; coverage is on the x-axis and reliability is on the y-axis. The ontology is denoted by the color of the disc, while the area of the disc reflects the frequency of the GO term in the 16-01-2008 GOA release. To be visualized in the plot, a GO term needs to have assigned at least 10 electronic/curated annotations in the 16-01-2008 GOA release, and at least 10 experimental annotations in the 11-01-2011 GOA release. The colored lines correspond to the mean values for the respective axes.

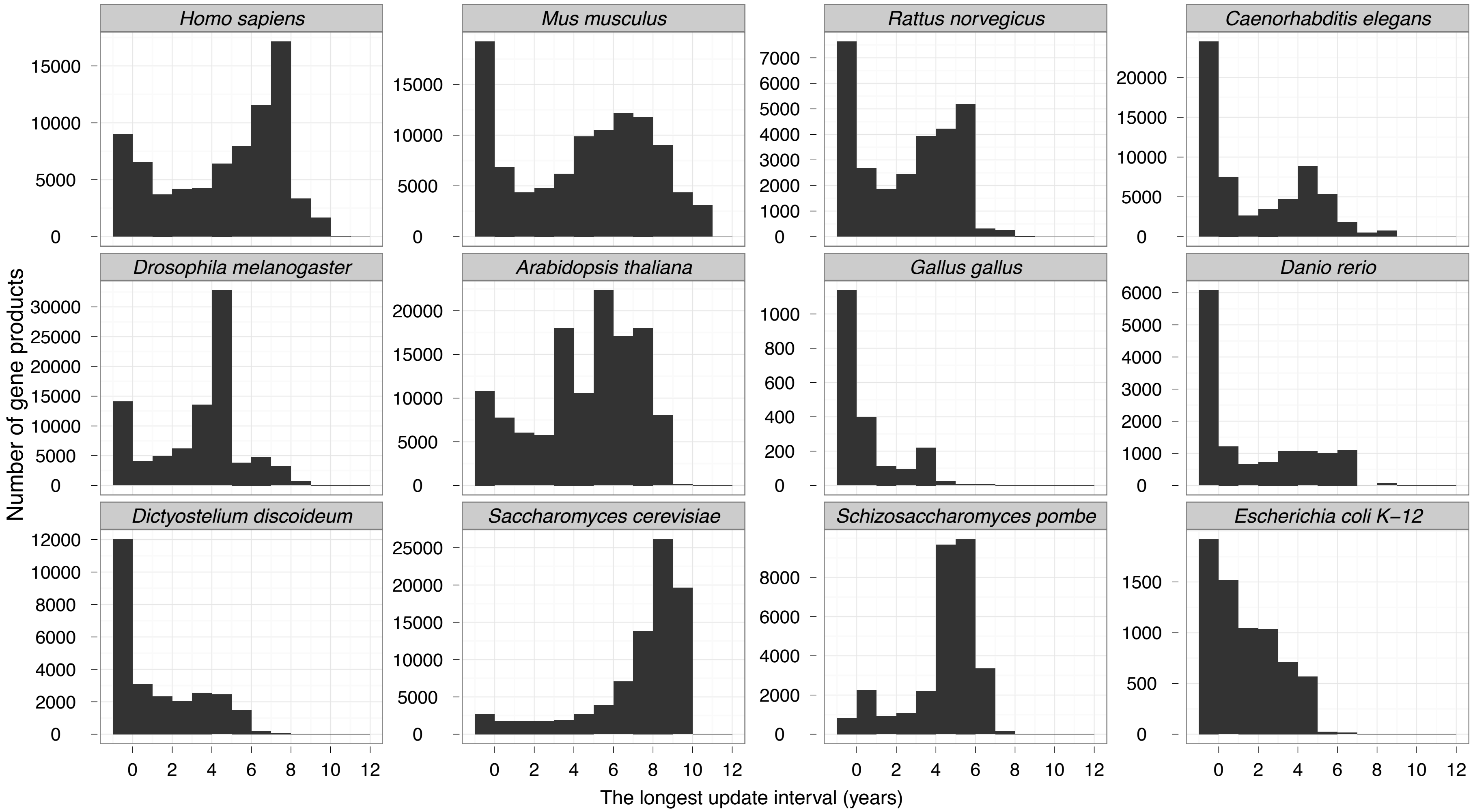

Figure S12. The span of curated annotation updates in the UniProt-GOA releases. Each panel summarizes the data for the model organism denoted in the header. The y-axis denotes the number of gene products with the longest annotation update interval denoted on the x-axis. The longest update interval is the time between the first and the last annotation assigned to one gene product.

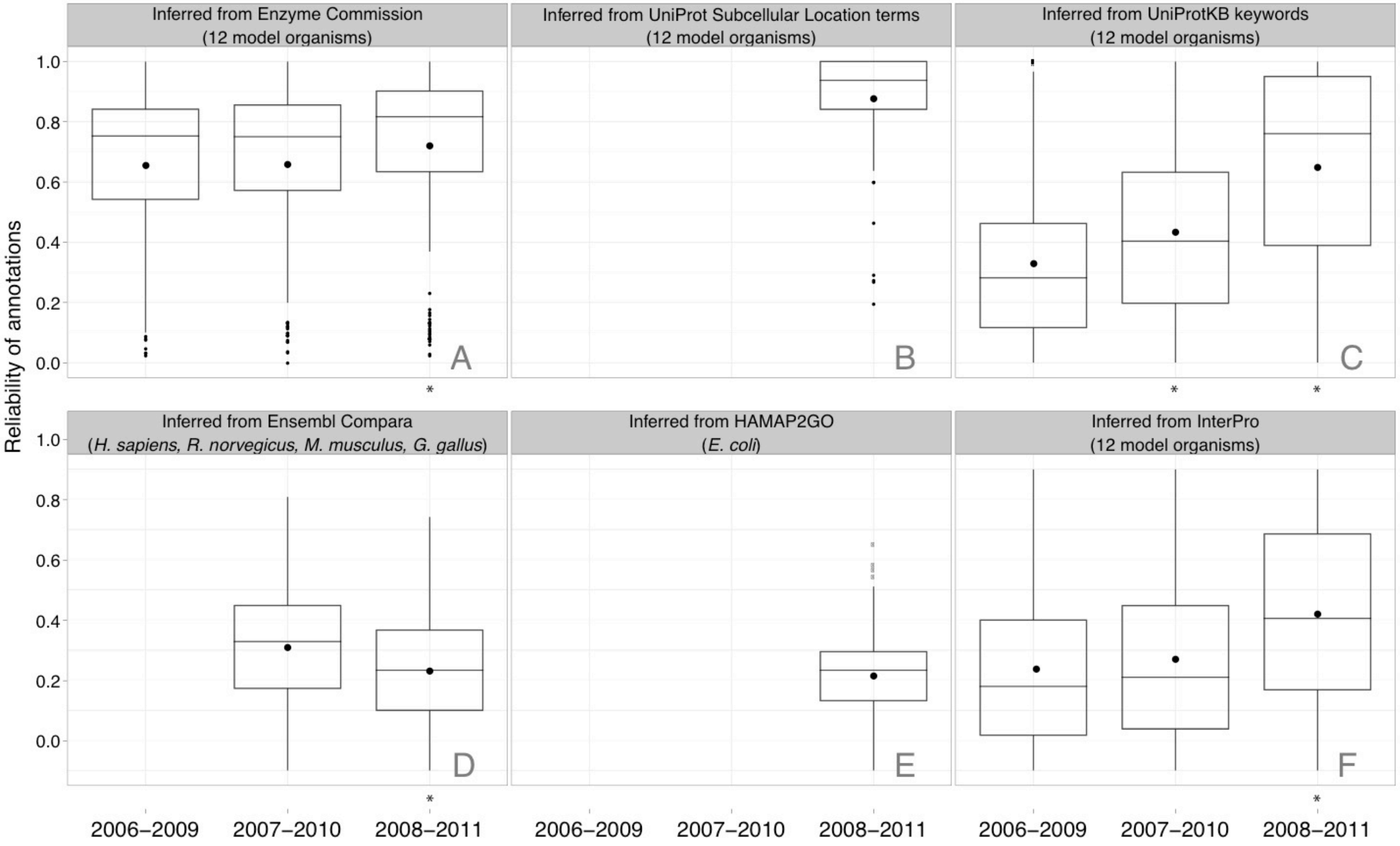

Figure S13. Summary statistics of reliability; each reference code is evaluated separately. The 12 model organisms are *Homo sapiens, Mus musculus, Rattus norvegicus, Caenorhabditis elegans, Drosophila melanogaster, Arabidopsis thaliana, Gallus gallus, Danio rerio, Dictyostelium discoideum, Saccharomyces cerevisiae, Schizosaccharomyces pombe*, and *Escherichia coli* K-12. Each boxplot summarizes the evaluation period indicated on the x axis: reliability of the 13-01-2006 GOA release, evaluated by the 21-01-2009 GOA release; reliability of the 19-01-2007 GOA release, evaluated by the 22-01-2010 GOA release; reliability of the 16-01-2008 GOA release, evaluated by the 11-01-2011 GOA release. Lower, mid, and upper horizontal lines denote the first quartile, median and the third quartile, respectively, while the black dot denotes the mean value. Values further than 1.5 interquartile range from the respective quartile are depicted by black points. An asterisk (*) below the boxplot denotes a significant difference of the median with respect to the previous interval, at a confidence level of 0.05 (Mann-Whitney U test, two-tailed).

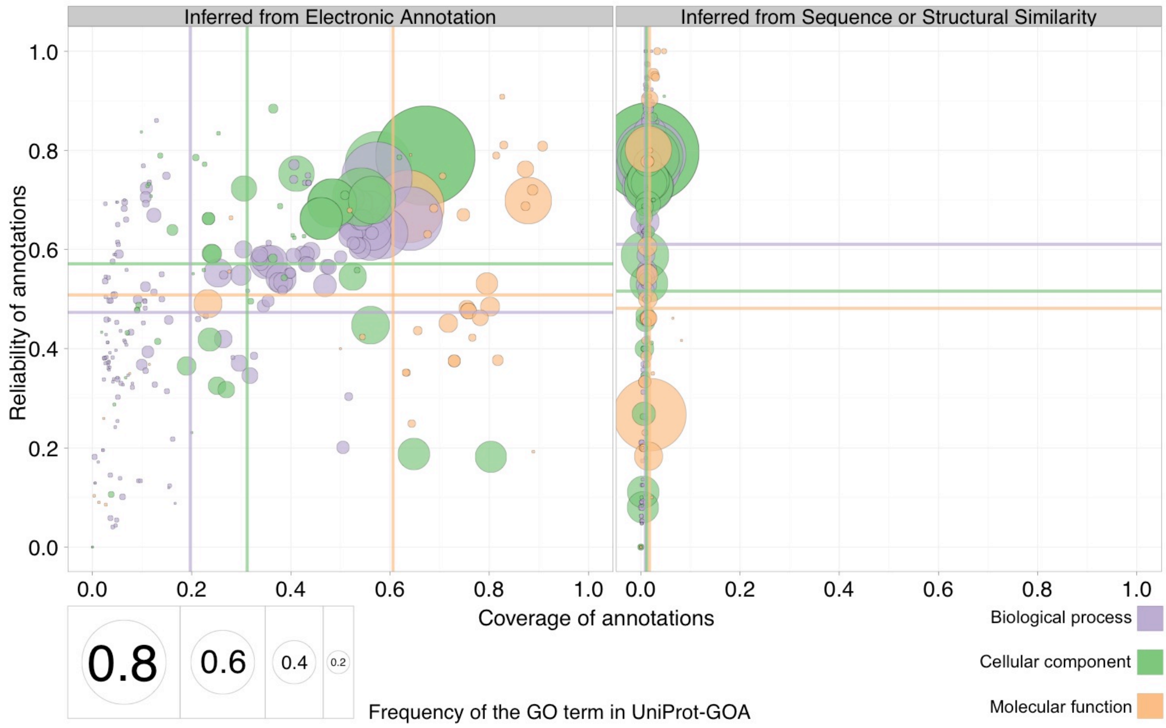

Figure S14. Quality of electronic annotations and annotations Inferred by Sequence Similarity (ISS) on a common set of GO terms. *Electronic* sources are inferred from InterPro, UniProt-GOA, UniProt-GOA (subcellular), Enzyme Commission, Ensembl Compara, and HAMAP. Quality of the 16-01-2008 GOA release is evaluated by the 11-01-2011 GOA release; coverage is on the x-axis and reliability is on the y-axis. The ontology is denoted by the color of the disc, while the area of the disc reflects the frequency of the GO term in the 16-01-2008 GOA release. To be visualized in the plot, a GO term needs to have assigned at least 10 electronic/curated annotations in the 16-01-2008 GOA release, and at least 10 experimental annotations in the 11-01-2011 GOA release. The colored lines correspond to the mean values for the respective axes.
